# Adherence to the cMIND and AIDD diets and their associations with anxiety in older adults in China

**DOI:** 10.3389/fnut.2025.1548072

**Published:** 2025-03-03

**Authors:** Yana Qi, Xinyu Xue, Ningsu Chen, Jie Gong, Dongyu Mu, Kai Zhao, Mengnan Zhao, Youping Li, Lei Shi, Jiajie Yu

**Affiliations:** ^1^Department of Clinical Nutrition, West China Hospital, Sichuan University, Chengdu, China; ^2^Chinese Evidence-Based Medicine Center, West China Hospital, Sichuan University, Chengdu, China

**Keywords:** cMIND diet, anti-inflammatory dietary diversity, anxiety, older, China

## Abstract

**Introduction:**

Anxiety is highly prevalent among older adults, and dietary interventions targeting nutrition may offer effective, practical strategies for preventing mental disorders. This study aimed to explore the association between the cMIND diet, anti-inflammatory dietary diversity (AIDD), and the risk of anxiety in older adults.

**Methods:**

A cross-sectional analysis was conducted using data from the 2018 Chinese Longitudinal Healthy Longevity Survey (CLHLS). Anxiety symptoms were assessed using the Generalized Anxiety Disorder (GAD-7) scale, while adherence to the cMIND diet and AIDD was evaluated through a food frequency questionnaire. Univariable and multivariable logistic regression analyses were performed to examine associations between dietary patterns and anxiety risk, with odds ratios (ORs) and 95% confidence intervals (CIs) reported. Random forest analysis was used to identify key factors influencing anxiety, and sensitivity analyses were conducted to test the robustness of the results.

**Results:**

A total of 13,815 participants aged 65 and older were included, with 1,550 (11.2%) identified with anxiety. Multivariable logistic models indicated that adherence to the cMIND diet or higher AIDD was associated with a 16–26% reduced risk of anxiety, with the adjusted ORs (95% CIs) for the cMIND diet ranging from 0.75 (0.64–0.87) to 0.75 (0.61–0.91), and for AIDD from 0.74 (0.62–0.88) to 0.84 (0.73–0.96). Sensitivity analyses confirmed the stability of these findings. Depression and sleep quality were identified as the most important factors contributing to anxiety, while diet was one of the few modifiable factors.

**Conclusion:**

This study provides evidence supporting the association between diet and anxiety in older adults, highlighting the potential of promoting healthy dietary patterns and targeted nutritional interventions as effective strategies for improving mental health in the aging population.

## 1 Introduction

Population aging is one of the most profound societal changes globally, raising significant concerns about the physical and mental health of older adults (1, 2). Anxiety disorders, characterized by inner tension, excessive fear in response to specific objects or situations, and anticipatory worry about adverse outcomes, are highly prevalent among older adults and often remain underdiagnosed and undertreated (3–5). Epidemiological studies in China have reported varying prevalence rates of anxiety in older populations, ranging from 4.7 to 20.8%, likely due to differences in study design and population heterogeneity (6). Anxiety not only contributes to chronic health conditions but is also associated with negative emotional states, such as pain and depression, further compounding its impact on overall health and well-being (7–9).

Conventional treatments for anxiety, such as psychotherapy and psychopharmacology, may not always be feasible or well-accepted among older adults in China, especially in contexts influenced by traditional beliefs, limited education, and economic constraints (10). Prolonged untreated anxiety can exacerbate the risk of physical disability and cognitive decline, emphasizing the need for alternative and accessible strategies to address this issue (11–13). In recent years, the field of nutritional psychiatry has gained momentum, with evidence suggesting that dietary interventions tailored to individual needs may offer a practical, cost-effective, and non-invasive approach to preventing and managing mental disorders (14).

Several dietary patterns, such as the Mediterranean diet and the Anti-Inflammatory Dietary Diversity Index (AIDDI) diet, have demonstrated potential in reducing anxiety risk (15). The Mediterranean-DASH Intervention for Neurodegenerative Delay (MIND) diet is a promising approach that combines elements of the Mediterranean and Dietary Approach to Stop Hypertension (DASH) diets while emphasizing neuroprotective benefits (16, 17), which has been modified to align with Chinese dietary habits by incorporating mushrooms, algae, garlic, and tea, while excluding butter, margarine, cheese, fried foods, red meat, and wine, and has been validated among older Chinese populations (16). These dietary patterns generally prioritize the intake of diverse, minimally processed foods rich in antioxidant and anti-inflammatory properties, such as fruits, vegetables, legumes, nuts, and tea. However, they differ in the specific food groups included and the scoring systems used to assess adherence (16, 18).

Over the past decade, growing evidence has highlighted the benefits of the MIND diet and AIDDI in improving cognitive function and reducing the risk of mental disorders (16, 18–20). However, high-quality studies examining their effects on anxiety risk remain limited, particularly among Chinese older adults. To address this gap, we utilized nationally representative data from the Chinese Longitudinal Health and Longevity Survey (CLHLS) to investigate the associations between adherence to the cMIND diet (Chinese version of MIND diet), anti-inflammatory dietary diversity, and anxiety risk in Chinese older adults. This study aims to provide evidence to support the development of individualized dietary recommendations and nutritional interventions for promoting mental health in aging populations.

## 2 Methods

### 2.1 Data source and participants

This cross-sectional study utilized data from the 2018 wave of the Chinese Longitudinal Health and Longevity Survey (CLHLS), a nationwide survey of adults aged 65 years and older across 23 of China’s 31 provinces, covering approximately 85% of the total population (21). The CLHLS employs a multi-stage, stratified cluster random sampling method with unequal proportions, ensuring a representative sample. The survey was initiated in 1998 with subsequent follow-ups in 2000, 2002, 2005, 2008–2009, 2011–2012, 2014, and 2017–2018. In 2018, the anxiety assessment scale was newly incorporated into the survey. Data were collected via structured, face-to-face interviews conducted by trained research assistants, covering demographic characteristics, physical and mental health, comorbidities, diet, lifestyle, and environmental factors. Details on data quality, including completeness, reliability, and validity, have been published previously (21, 22).

**Figure 1:**
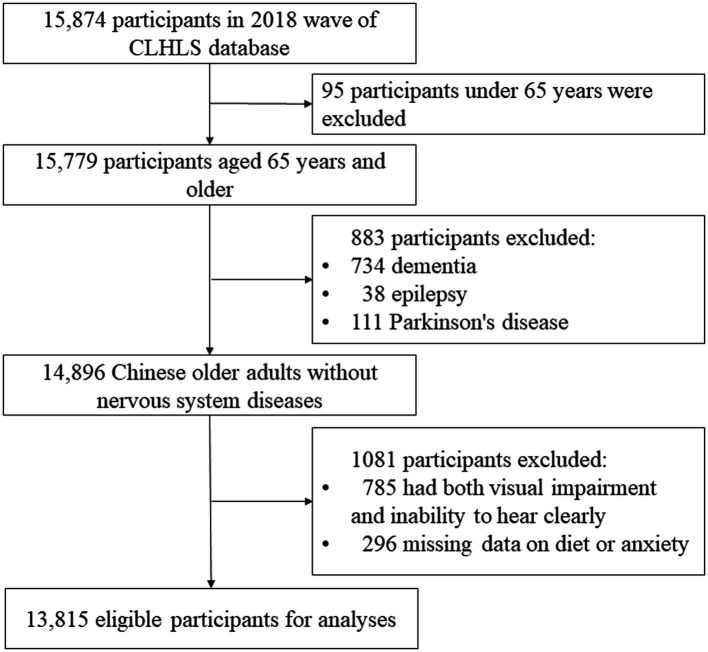
The flowchart of participants selection.

Participants aged below 65 years, those diagnosed with dementia, epilepsy, Parkinson’s disease, or other nervous system conditions that could confound the association between diet and anxiety, and those with dual visual and hearing impairments were excluded. Additionally, participants with missing data on key variables, including anxiety measures and dietary exposures, were excluded. Finally, a total of 13,815 eligible participants were included in this study (Figure 1).

### 2.2 Exposure

Dietary data were collected using food frequency questionnaires (FFQs), validated tools for assessing dietary intake (23–25). Trained interviewers gathered self-reported dietary information to calculate adherence to the cMIND diet and the anti-inflammatory dietary diversity (AIDD).

The cMIND diet was derived based on 11 food groups, including staple foods, fresh vegetables, mushrooms/algae, fresh fruits, vegetable/animal oils, fish, soybeans, garlic, nuts, tea, and sugar, as previously described (16). Each food group was assigned a score of 0, 0.5, or 1, except for staple foods and cooking oils, which were scored as 0 or 1. The total cMIND diet score ranged from 0 to 12, with higher scores indicating better adherence (Supplementary Table S1).

AIDD was assessed using the Dietary Inflammation Index (DII), which evaluates the anti-inflammatory potential of dietary intake based on 45 food parameters (26). In this study, AIDD focused on five key food groups: vegetables, fruits, legumes, nuts, and tea. Each food group consumed “almost every day” or “frequently (at least once per week)” was scored as 1; otherwise, it was scored as 0. The total AIDD score ranged from 0 to 5, with higher scores indicating greater diversity in anti-inflammatory foods (Supplementary Table S2).

For analysis, both the cMIND diet adherence score and AIDD scores were dichotomized into low and high scored groups based on the upper quartile cut-offs.

### 2.3 Outcome

Anxiety was assessed using the Generalized Anxiety Disorder (GAD-7) scale, a seven-item tool based on the DSM-IV criteria (the Diagnostic and Statistical Manual of Mental Disorders, Fourth Edition) (27) (Supplementary Table S3). Participants rated the frequency of anxiety symptoms over the past 2 weeks on a four-point scale (0–3). The total GAD-7 score ranged from 0 to 21, with higher scores indicating more severe symptoms. A score of ≥5 was used to define anxiety (6). The GAD-7 has been validated in Chinese populations, demonstrating good psychometric properties (28, 29). In this study, the Cronbach’s alpha coefficient for GAD-7 was 0.917 (95% CI: 0.912–0.921), indicating excellent internal consistency.

### 2.4 Covariates

To address potential confounding, we adjusted for a range of covariates selected based on previous studies and clinical relevance. These included demographic characteristics (e.g., region, age, sex, and ethnicity), socioeconomic factors (e.g., education, occupation, marital status, household registration, living arrangements, indoor air pollution, economic status, and pension insurance), lifestyle factors (e.g., smoking, drinking, and physical activity), health conditions (e.g., BMI, waist circumference, and comorbidities), functional status (e.g., frailty, cognitive impairment, activities of daily living, falls, and health change), psychological factors (e.g., sleep quality, depression, and quality of life), and social engagement (e.g., solitary and social activities).

### 2.5 Statistical analyses

Missing data were assessed for proportion and mechanism, and multiple imputations using random forests were performed to handle missing values. Baseline characteristics of participants were compared between low and high adherence groups for the cMIND diet and AIDD using frequencies (percentages) for categorical variables and means (standard deviations, SD) for continuous variables. Distribution differences were evaluated using Chi-square tests or Student’s *t*-tests.

Univariable and multivariable logistic regression analyses were conducted to assess the associations between dietary patterns and anxiety risk. Multilevel models with pure intercepts confirmed the absence of data clustering across regions. Four models were constructed for each dietary exposure:

- **Model 1:** Adjusted for 26 demographic, socioeconomic, lifestyle, and health-related variables.
- **Model 2:** Further adjusted for sleep quality and quality of life.
- **Model 3:** Additionally adjusted for depression, given its potential influence on diet-anxiety associations.
- **Model 4:** Included significant interaction terms (A × B) identified in Model 3.

Adjusted odds ratios (aORs) and 95% confidence intervals (CIs) were reported. Model performance was evaluated using the Akaike Information Criterion (AIC), and collinearity was assessed with Generalized Variance Inflation Factors (GVIF). Outcome events per variable (EPV) were checked to ensure model robustness.

Random forest analysis was used to identify the relative importance of dietary factors compared to other predictors of anxiety. Sensitivity analyses included: (1) treating dietary scores as continuous variables; (2) analyzing the original dataset without imputation; and (3) adjusting models using sampling weights based on the 2020 National Census Data to ensure population representativeness.

All statistical analyses were conducted using R software (version 4.1.1). A two-tailed *p*-value <0.05 was considered statistically significant.

## 3 Results

A total of 13,815 Chinese adults aged 65 years and older were included in the analysis after excluding 883 ineligible participants and 1,081 records due to missing or incomplete data. The flowchart of participant selection is presented in Figure 1. Compared to those excluded (12.4%, 1,964/15,874), the included participants exhibited distinct baseline characteristics (Supplementary Table S4). Missing data were determined to be missing at random, as exclusion was associated with other observed variables (data not shown).

Table 1 summarizes the baseline characteristics of included participants. Among them, 11.2% were identified with anxiety. The mean age was 84.7 years (weighted age: 76.2 years), and 54.9% were female. The prevalence of cardiometabolic diseases was 52.1%, frailty index >0.25 was 15.3%, cognitive impairment was 19.1%, poor sleep quality was 14.7%, and depression was 14.9%. High adherence to the cMIND diet and the anti-inflammatory dietary diversity (AIDD) was observed in 27.0% (3,724/13,815) and 37.4% (5,169/13,815) of participants, respectively. Significant differences in most baseline characteristics were observed between low and high adherence groups for both dietary patterns, except for the prevalence of respiratory or digestive system diseases.

**Table 1:** Characteristics of participants and distribution between low vs. high scored dietary patterns.

Characteristics	Overall	Adherence to cMIND		Anti-inflammatory DD	
		Low	High	Low	High
	(*n* = 13,815)	(*n* = 10,091)	(*n* = 3,724)	(*n* = 8,646)	(*n* = 5,169)
Anxiety					
GAD-7 score: mean (SD)	1.34 (2.71)	1.48 (2.88)	0.97 (2.16)	1.54 (2.93)	1.01 (2.26)
GAD-7 score < 5	12,265 (88.8)	8,824 (87.4)	3,441 (92.4)	7,507 (86.8)	4,758 (92.0)
GAD-7 score ≥ 5	1,550 (11.2)	1,267 (12.6)	283 (7.6)	1,139 (13.2)	411 (8.0)
Regions					
North	822 (6.0)	411 (4.1)	411 (11.0)	267 (3.1)	555 (10.7)
Northeast	612 (4.4)	283 (2.8)	329 (8.8)	223 (2.6)	389 (7.5)
East	5,595 (40.5)	3,879 (38.4)	1716 (46.1)	3,273 (37.9)	2,322 (44.9)
Central South	5,027 (36.4)	4,127 (40.9)	900 (24.2)	3,679 (42.6)	1,348 (26.1)
West	1759 (12.7)	1,391 (13.8)	368 (9.9)	1,204 (13.9)	555 (10.7)
Age (years)					
65 ~ 79	5,034 (36.4)	3,204 (31.8)	1830 (49.1)	2,865 (33.1)	2,169 (42.0)
80 ~ 89	3,661 (26.5)	2,705 (26.8)	956 (25.7)	2,354 (27.2)	1,307 (25.3)
90 ~ 99	3,000 (21.7)	2,370 (23.5)	630 (16.9)	1950 (22.6)	1,050 (20.3)
≥100	2,120 (15.3)	1812 (18.0)	308 (8.3)	1,477 (17.1)	643 (12.4)
Sex					
Male	6,230 (45.1)	4,175 (41.4)	2055 (55.2)	3,586 (41.5)	2,644 (51.2)
Female	7,585 (54.9)	5,916 (58.6)	1,669 (44.8)	5,060 (58.5)	2,525 (48.8)
Ethnicity: Han	11,264 (81.5)	8,072 (80.0)	3,192 (85.7)	6,887 (79.7)	4,377 (84.7)
Educated time (year)					
0 (Illiteracy)	6,655 (48.2)	5,571 (55.2)	1,084 (29.1)	4,775 (55.2)	1880 (36.4)
1 ~ 6	4,512 (32.7)	3,223 (31.9)	1,289 (34.6)	2,838 (32.8)	1,674 (32.4)
7 ~ 9	1,374 (9.9)	727 (7.2)	647 (17.4)	606 (7.0)	768 (14.9)
10 ~ 12	800 (5.8)	372 (3.7)	428 (11.5)	297 (3.4)	503 (9.7)
≥13	474 (3.4)	198 (2.0)	276 (7.4)	130 (1.5)	344 (6.7)
Agriculture-based occupation	7,236 (52.4)	5,880 (58.3)	1,356 (36.4)	5,195 (60.1)	2041 (39.5)
Marital status					
Married, living with spouse	5,744 (41.6)	3,676 (36.4)	2068 (55.5)	3,245 (37.5)	2,499 (48.3)
Widow	7,664 (55.5)	6,121 (60.7)	1,543 (41.4)	5,140 (59.4)	2,524 (48.8)
Divorce or separation	294 (2.1)	203 (2.0)	91 (2.4)	184 (2.1)	110 (2.1)
Never married	113 (0.8)	91 (0.9)	22 (0.6)	77 (0.9)	36 (0.7)
Household registration					
Urban	3,870 (28.0)	2,123 (21.0)	1747 (46.9)	1,615 (18.7)	2,255 (43.6)
Rural	9,945 (72.0)	7,968 (79.0)	1977 (53.1)	7,031 (81.3)	2,914 (56.4)
Living arrangements					
Living with family	11,052 (80.0)	7,920 (78.5)	3,132 (84.1)	6,790 (78.5)	4,262 (82.5)
Living alone	2,281 (16.5)	1857 (18.4)	424 (11.4)	1,621 (18.7)	660 (12.8)
Collective institutions	482 (3.5)	314 (3.1)	168 (4.5)	235 (2.7)	247 (4.8)
Indoor air pollution^a^					
None	8,205 (59.4)	5,571 (55.2)	2,634 (70.7)	4,687 (54.2)	3,518 (68.1)
Level 1	4,824 (34.9)	3,816 (37.8)	1,008 (27.1)	3,320 (38.4)	1,504 (29.1)
Level 2	786 (5.7)	704 (7.0)	82 (2.2)	639 (7.4)	147 (2.8)
Economic status^b^					
Rich	2,725 (19.7)	1,659 (16.4)	1,066 (28.6)	1,312 (15.2)	1,413 (27.3)
Medium	9,674 (70.0)	7,199 (71.3)	2,475 (66.5)	6,190 (71.6)	3,484 (67.4)
Poor	1,416 (10.2)	1,233 (12.2)	183 (4.9)	1,144 (13.2)	272 (5.3)
Pension insurance participation	4,999 (36.2)	3,788 (37.5)	1,211 (32.5)	3,210 (37.1)	1789 (34.6)
Smoking					
Never	9,434 (68.3)	7,084 (70.2)	2,350 (63.1)	6,070 (70.2)	3,364 (65.1)
Previous	2076 (15.0)	1,364 (13.5)	712 (19.1)	1,153 (13.3)	923 (17.9)
Current	2,305 (16.7)	1,643 (16.3)	662 (17.8)	1,423 (16.5)	882 (17.1)
Drinking					
Never	10,188 (73.7)	7,609 (75.4)	2,579 (69.3)	6,495 (75.1)	3,693 (71.4)
Previous	1,598 (11.6)	1,149 (11.4)	449 (12.1)	1,023 (11.8)	575 (11.1)
Current	2029 (14.7)	1,333 (13.2)	696 (18.7)	1,128 (13.0)	901 (17.4)
Regular physical activity	3,380 (24.5)	1969 (19.5)	1,411 (37.9)	1,644 (19.0)	1736 (33.6)
BMI (kg/m^2^)					
<18.5 (Underweight)	2,283 (16.5)	1903 (18.9)	380 (10.2)	1,626 (18.8)	657 (12.7)
18.5 ~ 24.9 (Normal)	8,315 (60.2)	6,121 (60.7)	2,194 (58.9)	5,238 (60.6)	3,077 (59.5)
25.0 ~ 29.9 (Overweight)	2,690 (19.5)	1705 (16.9)	985 (26.5)	1,460 (16.9)	1,230 (23.8)
≥30.0 (Obese)	527 (3.8)	362 (3.6)	165 (4.4)	322 (3.7)	205 (4.0)
WC > 85 cm (male); >80 cm (female)	7,400 (53.6)	5,094 (50.5)	2,306 (61.9)	4,373 (50.6)	3,027 (58.6)
Comorbidities					
Visual impairment	2,135 (15.5)	1775 (17.6)	360 (9.7)	1,516 (17.5)	619 (12.0)
Hearing impairment	5,219 (37.8)	4,093 (40.6)	1,126 (30.2)	3,495 (40.4)	1724 (33.4)
Toothache or cheek pain^*^	2,426 (17.6)	1,676 (16.6)	750 (20.1)	1,537 (17.8)	889 (17.2)
Cardiometabolic diseases^c^	7,204 (52.1)	4,902 (48.6)	2,302 (61.8)	4,241 (49.1)	2,963 (57.3)
Respiratory system diseases^d,**^	1,428 (10.3)	1,020 (10.1)	408 (11.0)	878 (10.2)	550 (10.6)
Digestive system diseases^e,**^	1,066 (7.7)	758 (7.5)	308 (8.3)	648 (7.5)	418 (8.1)
Immune system diseases^f,*^	1819 (13.2)	1,290 (12.8)	529 (14.2)	1,105 (12.8)	714 (13.8)
Cancer	179 (1.3)	106 (1.1)	73 (2.0)	91 (1.1)	88 (1.7)
Frailty index >0.25	2,107 (15.3)	1,691 (16.8)	416 (11.2)	1,405 (16.3)	702 (13.6)
Cognitive impairment^g^	2,641 (19.1)	2,184 (21.6)	457 (12.3)	1853 (21.4)	788 (15.2)
Fall last year	3,070 (22.2)	2,357 (23.4)	713 (19.1)	2065 (23.9)	1,005 (19.4)
Health change last year^h^					
Stable	7,060 (51.1)	5,066 (50.2)	1994 (53.5)	4,289 (49.6)	2,771 (53.6)
Better	1808 (13.1)	1,197 (11.9)	611 (16.4)	1,018 (11.8)	790 (15.3)
Worse	4,947 (35.8)	3,828 (37.9)	1,119 (30.0)	3,339 (38.6)	1,608 (31.1)
Sleep quality^i^					
Good	7,309 (52.9)	5,039 (49.9)	2,270 (61.0)	4,227 (48.9)	3,082 (59.6)
Neutral	4,470 (32.4)	3,416 (33.9)	1,054 (28.3)	2,953 (34.2)	1,517 (29.3)
Poor	2036 (14.7)	1,636 (16.2)	400 (10.7)	1,466 (17.0)	570 (11.0)
Quality of life^j^					
Good	9,807 (71.0)	6,837 (67.8)	2,970 (79.8)	5,772 (66.8)	4,035 (78.1)
Neutral	3,637 (26.3)	2,921 (28.9)	716 (19.2)	2,569 (29.7)	1,068 (20.7)
Poor	371 (2.7)	333 (3.3)	38 (1.0)	305 (3.5)	66 (1.3)
Depression					
CESD-10 score < 12	11,754 (85.1)	8,385 (83.1)	3,369 (90.5)	7,130 (82.5)	4,624 (89.5)
CESD-10 score ≥ 12	2061 (14.9)	1706 (16.9)	355 (9.5)	1,516 (17.5)	545 (10.5)
BADL score^k^, mean (SD)	6.91 (2.24)	7.01 (2.36)	6.64 (1.82)	6.95 (2.30)	6.84 (2.12)
IADL score^l^, mean (SD)	13.31 (5.94)	13.88 (6.02)	11.77 (5.42)	13.73 (5.95)	12.62 (5.86)
Participating solitary activity^m^	11,566 (83.7)	8,119 (80.5)	3,447 (92.6)	6,971 (80.6)	4,595 (88.9)
Participating social activity^n,*^	13,459 (97.4)	9,807 (97.2)	3,652 (98.1)	8,422 (97.4)	5,037 (97.4)

BADL, basic activities of daily living; BMI, body mass index; CESD, center for epidemiologic studies depression scale; DD, dietary diversity; GAD, the generalized anxiety disorder scale; IADL, instrumental activity of daily living; SD, standard deviation; WC, waist circumference.

Data were shown as absolute frequency and percentage, unless otherwise specified. The criteria for high adherence to the cMIND and AIDD diets were defined as scores exceeding the upper quartile cut-off, while scores at or below this threshold were classified as low adherence.

Chi-square or *t*-test: differences between diet groups were significant (*p* < 0.05), unless otherwise specified. ^*^Anti-inflammatory DD: *p* > 0.05; ^**^Adherence to cMIND and Anti-inflammatory DD both *p* > 0.05.

^a^Indoor air pollution: defined 0 ~ 2 levels by indoor moldy taste and cooking with polluting fuels (e.g., coal, wood, or kerosene).

^bEconomic status was categorized as “rich,” “medium,” or “poor” based on the interviewee’s self-assessment relative to others, with “rich” for “very rich” or “rich,” “medium” for “so-so,” and “poor” for “poor” or “very poor”.^

^cCardiometabolic diseases included hypertension, diabetes, heart diseases, cerebrovascular diseases or stroke, and dyslipidemia.^

^dRespiratory system diseases included bronchopneumonia, emphysema, asthma, and tuberculosis.^

^eDigestive system diseases included gastrointestinal ulcer, cholecystitis, cholelithiasis, and hepatitis.^

^fImmune system diseases included arthritis and rheumatism.^

^g^Cognitive impairment was identified by the Chinese version of 30-point mini-mental state examination (MMSE) about orientation, memory, attention, calculation, language, and written and visual construction, which defined those scored less than or equal to 17 as cognitive impairment for those without formal education, less than or equal to 20 for those with 1–6 years of education, and less than or equal to 24 for those with more than 6 years of education.

^hHealth change was categorized as “stable” if the interviewee rated their health as “almost the same” compared to 1 year ago, “better” if rated as “much better” or “slightly better,” and “worse” if rated as “much worse” or “slightly worse”.^

^iSleep quality was categorized as “good” if the interviewee rated their sleep quality as “good” or “very good,” “neutral” if rated as “so-so,” and “poor” if rated as “poor” or “very poor”.^

^jQuality of life was categorized as “good” if the interviewee rated their quality of life as “good” or “very good,” “neutral” if rated as “so-so,” and “poor” if rated as “poor” or “very poor”.^

^kBADL included bathing, dressing, toileting, indoor moving, continence of defecation, and eating, and total scored 6–18 with larger value indicating poorer activities.^

^lIADL included visiting neighbors, shopping, cooking, washing, continuously walking two kilometers, lifting 5 kilograms objects, squat and stand up three times, or taking public transportation by themselves, and total scored 8–24 with larger value indicating poorer activities.^

^mSolitary activity included reading newspapers or books, watching TV or listening to radio, doing housework, gardening, and keeping pets or domestic animals.^

^nSocial activity included playing cards or mahjong, attending social activities, participating in outdoor activities, regular exercise, or travelling at least once in the past 2 years.^

The mean anxiety scores differed significantly between low and high adherence groups for both dietary patterns (cMIND diet: 1.48 vs. 0.97; AIDD: 1.54 vs. 1.01; both *t*-tests: *p* < 0.05). Spearman correlation analyses revealed significant negative correlations between anxiety and both dietary scores (Supplementary Figures S1, S2). Univariable logistic regression further confirmed significant differences in anxiety prevalence between low and high adherence groups for both diets (Supplementary Table S5).

Multivariable logistic regression models demonstrated that high adherence to the cMIND diet and AIDD was significantly associated with a lower risk of anxiety (Table 2). All four models showed consistent results, with significant interaction effects between diet and depression (interaction term in Model 3: *p* < 0.05). Model 4, which included all variables and the interaction term, exhibited the best model fit with the lowest Akaike Information Criterion (AIC) value (Supplementary Table S6). The adjusted odds ratios (ORs) and 95% confidence intervals (CIs) for high adherence to the cMIND diet across Models 1–4 were 0.75 (0.64–0.87), 0.82 (0.70–0.96), 0.84 (0.71–0.99), and 0.75 (0.61–0.91), respectively. For high adherence to AIDD, the corresponding ORs were 0.75 (0.66–0.86), 0.82 (0.72–0.94), 0.84 (0.73–0.96), and 0.74 (0.62–0.88) (Table 2).

**Table 2:** Multivariable logistic regression of adherence to the cMIND diet, anti-inflammatory dietary diversity and risk of anxiety.

Dietary patterns	Model 1	Model 2	Model 3	Model 4
	aOR (95%CI)	aOR (95%CI)	aOR (95%CI)	aOR (95%CI)
Adherence to the cMIND diet				
Main analysis^*^	0.75 (0.64–0.87)	0.82 (0.70–0.96)	0.84 (0.71–0.99)	0.75 (0.61–0.91)
Sensitivity analysis 1	0.90 (0.86–0.94)	0.94 (0.90–0.98)	0.95 (0.91–0.99)	0.91 (0.87–0.97)
Sensitivity analysis 2^*^	0.70 (0.58–0.83)	0.77 (0.64–0.93)	0.80 (0.66–0.97)	0.67 (0.54–0.86)
Sensitivity analysis 3^*^	0.96 (0.84–1.08)	1.05 (0.92–1.19)	1.05 (0.92–1.20)	0.94 (0.80–1.10)
Anti-inflammatory dietary diversity				
Main analysis ^*^	0.75 (0.66–0.86)	0.82 (0.72–0.94)	0.84 (0.73–0.96)	0.74 (0.62–0.88)
Sensitivity analysis 1	0.85 (0.80–0.90)	0.91 (0.85–0.97)	0.92 (0.86–0.98)	0.85 (0.78–0.93)
Sensitivity analysis 2^*^	0.70 (0.60–0.82)	0.76 (0.65–0.90)	0.79 (0.67–0.93)	0.69 (0.56–0.85)
Sensitivity analysis 3^*^	0.74 (0.66–0.83)	0.78 (0.69–0.88)	0.79 (0.69–0.90)	0.77 (0.66–0.90)

^*^The adjusted odds ratio (OR) and 95% confidence interval (CI) were calculated of the high scored dietary pattern comparing with the low scored group.

Model 1 adjusted for regions, age, sex, ethnicity, educated time, occupation, marital status, household registration, living arrangements, indoor air pollution, economic status, pension insurance, smoking, drinking, physical activity, BMI, waist circumference, toothache or cheek pain, cardiometabolic diseases, respiratory system diseases, digestive system diseases, immune system diseases, cancer, frailty, fall, and health change last year.

Model 2 adjusted for the same confounders as model 1, added sleep quality and quality of life.

Model 3 adjusted for the same confounders as model 2, added depression.

Model 4 adjusted for the same confounders as model 3, added the interaction term between diet and depression.

Sensitivity analysis 1 included the continuous score for dietary patterns without grouping.

Sensitivity analysis 2 were fitted using the original sample before multiple imputation.

Sensitivity analysis 3 were weighted based on the distribution of age and sex across included 23 provinces from the National Census Data in 2020.

Sensitivity analyses supported the robustness of these findings. Multivariable models using continuous dietary scores yielded consistent associations, albeit with varied effect sizes. Analyses using the original dataset without imputation produced more significant results. Weighted models based on the national census data showed stable results for AIDD, whereas associations for the cMIND diet were not statistically significant (Table 2).

**Figure 2:**
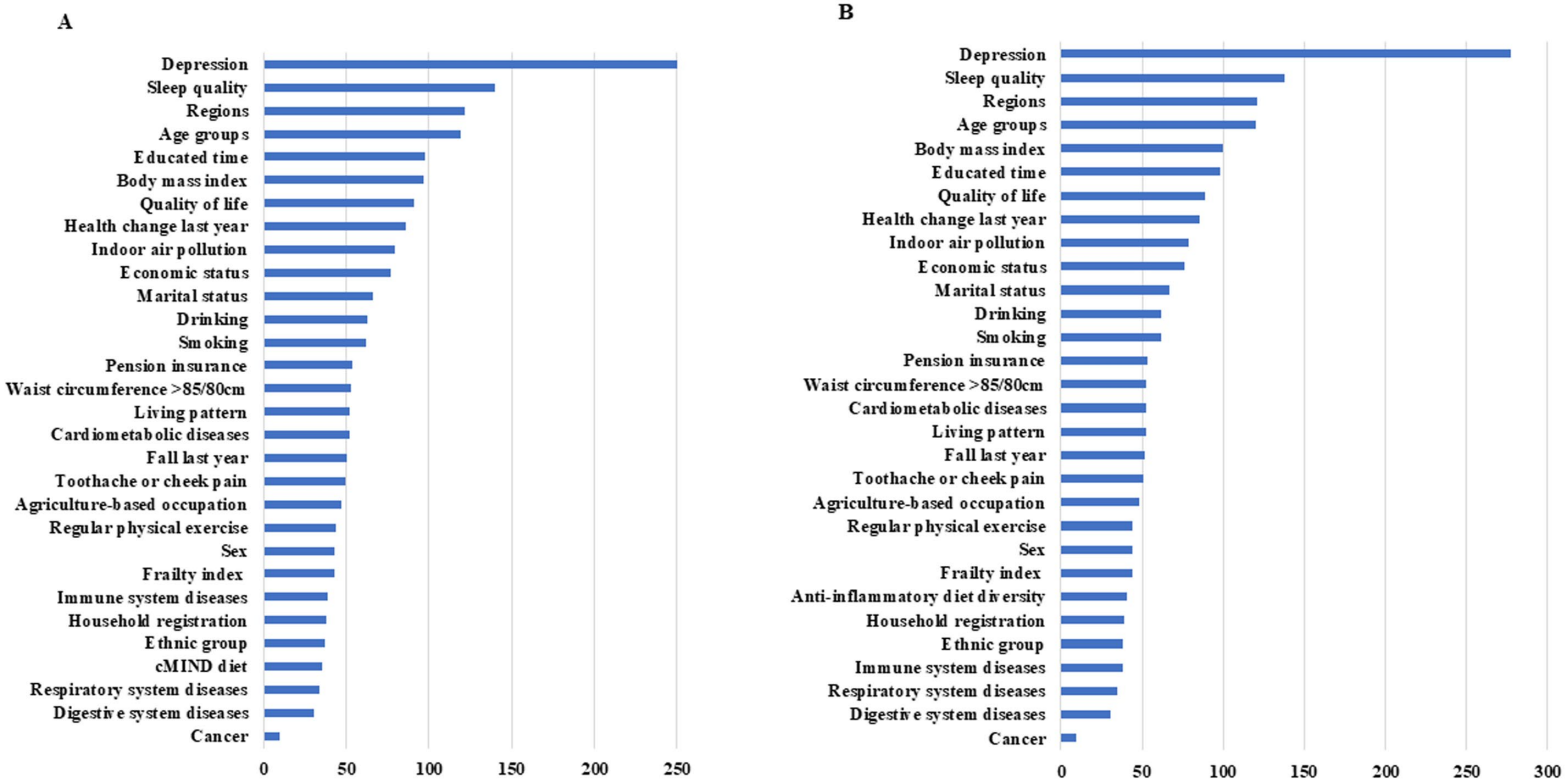
Importance of factors associated with the risk of anxiety The importance rank was determined by decrease in GINI coefficient after variable replacement and larger value indicates more importance; **(A)** (left) for the cMIND diet and **(B)** (right) for anti-inflammatory dietary diversity.

Random forest analysis ranked diet as a low-to-moderate factor influencing anxiety risk. Nonetheless, it remained one of the few modifiable factors, following indoor air pollution, drinking, smoking, and regular physical activity. Depression and sleep quality emerged as the most critical factors associated with anxiety (Figure 2).

## 4 Discussion

Our study found a significant association between high adherence to the cMIND diet and anti-inflammatory dietary diversity with a reduced risk of anxiety in Chinese older adults, even after adjusting for various confounders. This provides evidence supporting the promotion of healthy dietary patterns and the implementation of targeted nutritional interventions to reduce anxiety risk. Diet, being one of the few modifiable factors, is more likely to be accepted by older adults, making it a practical and sustainable approach. Additionally, depression and sleep quality were identified as the most important factors related to anxiety, suggesting the necessity of jointly addressing these factors due to their potential shared pathogenic causes.

Anxiety, one of the most common psychiatric disorders, results from complex interactions among genetic, epigenetic, and environmental factors. The effect of diet on anxiety can be attributed to various bioactive dietary compounds (30) and the synergistic or antagonistic interactions of phytochemicals or nutrients at chemical, biochemical, and physiological levels (31, 32). These mechanisms involve multiple interlinked biological pathways (14), with inflammation serving as a critical mediator. Recently, attention has focused on the gut-brain axis as another important pathway (33).

To explain the cMIND diet’s effect on anxiety, we examined potential evidence for each component of the diet (34, 35). The cMIND diet recommends an adequate intake of staple foods, fresh vegetables and fruits, vegetable oils, mushrooms/algae, fish, soybeans, garlic, nuts, and tea, while discouraging sugar/sweets (16). Previous studies have demonstrated that fresh vegetables and fruits are rich sources of folate, vitamin C, carotenoids, and flavonoids (34, 35). Vegetable oils provide unsaturated fatty acids that benefit brain health and have antioxidative and anti-inflammatory effects (36). Mushrooms inhibit the production of amyloid-β and phosphorylated tau protein, while promoting neurite outgrowth and nerve growth factor synthesis (37). Fish, especially deep-sea varieties rich in omega-3 fatty acids, play an essential role in neuronal membranes (38). Soybeans are a source of essential amino acids, soy protein, and isoflavones, all of which have anti-inflammatory and antioxidant properties (39). Garlic contains compounds such as alliin, allicin, and gamma-glutamyl cysteine, which modulate reactive oxygen species (ROS) (40). Nuts are packed with vitamins B and E, minerals, flavonoids, and high levels of unsaturated fatty acids, which reduce inflammation and oxidative stress (41). Tea polyphenols exert neuroprotective effects through antioxidant activity, iron chelation, and modulation of signal transduction. In contrast, sugar/sweets can disrupt glucose and insulin metabolism, leading to neuroinflammation and oxidative stress, and causing structural changes in the brain (42).

The effect of anti-inflammatory dietary diversity on anxiety can also be explained using the evidence above. Furthermore, it can be interpreted from the perspective of the gastrointestinal microbiome. Nutrients derived from dietary intake interact with gut microbiota, which convert them into absorbable substances in the gut (43). Gut microbes also release signaling molecules that interact with the immune system, stimulating innate and adaptive immune functions (43). Disruption of the gut microbiota can disturb the balance between nutrients and immune responses (43), leading to chronic inflammation. Thus, diets rich in anti-inflammatory foods, or cMIND diet containing multiple anti-inflammatory foods, have the potential to improve micronutrient absorption and regulate immune responses by promoting healthy gut microbiota (14, 43).

To date, clinical evidence on the association between the MIND diet or anti-inflammatory dietary diversity and anxiety remains scarce, particularly in older populations. A cross-sectional multicenter study conducted in Iran among 3,176 adults found that higher adherence to the MIND diet was associated with a lower risk of depression and psychological distress, though no significant association was observed with anxiety (44). Other studies have identified associations between dietary diversity and anxiety in women (45, 46) or anxiety related to COVID-19 (47). Compared to these studies, our research focused specifically on older adults, a population more prone to anxiety, and utilized localized dietary measurement tools adapted for the Chinese context (16). Moreover, we comprehensively adjusted for confounding factors such as sleep quality, economic status, and comorbidities. Although our study did not adjust for energy intake due to data limitations, prior research has shown that energy intake is not significantly associated with anxiety after accounting for dietary patterns, suggesting minimal impact on our conclusions (48).

Of the related factors, few studies have investigated modifiable risk factors for anxiety, potentially due to their subtle or non-significant effects (49). However, identifying these factors provides insight into cost-effective and safe approaches (50, 51), such as dietary improvements or nutritional interventions, to reduce the prevalence and burden of anxiety in the long term. In our study, depression and sleep quality emerged as key factors for anxiety. Anxiety and depression often co-occur, likely due to shared pathogenic mechanisms such as chronic stress and overlapping negative emotional states (52). Network analyses have demonstrated stable connections between depressive and anxiety symptoms, with “sadness” serving as a central symptom in the psychopathology network (53). Sleep disturbances, characterized by dissatisfaction with sleep quality, are predictors of mental disorders, including anxiety (54, 55). Although the relationship between insomnia and anxiety may be bidirectional, insomnia symptoms often precede anxiety (56). Compared to demographic or social determinants, depression and sleep quality are more direct and proximal causes of anxiety, suggesting that joint interventions targeting these factors could yield substantial benefits.

Our study has several strengths. First, it provides additional evidence on the association between diet and anxiety in older adults, offering a foundation for designing specific healthy dietary patterns tailored to individual conditions, such as comorbidities. Combining certain foods or increasing specific nutrients could enhance dietary effects. Such dietary interventions have been shown to be cost-effective for improving multiple health outcomes and quality of life (50, 51). Second, we rigorously adjusted for confounding factors using various analytical strategies that incorporated all available data, reducing residual confounding. We also conducted sensitivity analyses to confirm the robustness of our results and adjusted for sampling weights to ensure population-representative estimates. To our knowledge, this is the first study using CLHLS data to adjust for sampling weights in such analyses.

Nonetheless, this study has limitations. First, its cross-sectional design restricts causal inferences, and the exclusion of ineligible participants limits its generalizability to all Chinese older adults. Second, there is a lack of universally accepted and validated tools for assessing dietary quality, and the validity and reliability of the measurement tools used in this study remain uncertain. The association between the cMIND diet and anxiety may have become insignificant in weighted analyses due to discrepancies between the population distribution in the 2020 National Census and 2018 CLHLS data, or due to limitations in the dietary measurement tools. Prospective studies are needed to confirm these findings and further explore the underlying mechanisms.

## 5 Conclusion

In conclusion, adherence to the cMIND diet or high anti-inflammatory dietary diversity appears to be a promising approach for reducing anxiety in Chinese older adults. These findings highlight the potential of dietary patterns as modifiable and accessible strategies to promote mental health in aging populations. Incorporating evidence-based dietary recommendations and nutritional interventions into public health policies and individual care plans could play a vital role in preventing and managing anxiety, ultimately enhancing overall quality of life in older adults.

## Data Availability

The original contributions presented in the study are included in the article/Supplementary material, further inquiries can be directed to the corresponding authors.
