# Supplementary material for: Adherence to the cMIND and AIDD diets and their associations with anxiety in older adults in China
Source: Front Nutr. 2025 Mar 3;12:1548072. doi: 10.3389/fnut.2025.1548072 (PMC11911210; doi:10.3389/fnut.2025.1548072)
Supplement: Supplementary file 1 [file Data_Sheet_1.docx]

# Supplementary Figures and Tables

## Table S1. The components and scores of the cMIND diet

| **Components** | **Score 0** | **Score 0.5** | **Score 1** |
| --- | --- | --- | --- |
| Type of staple food | Rice/wheat |  | Whole grains |
| Amount of staple food | <250g or >400g |  | 250-400g |
| Fresh vegetables | ≤2 servings/week | 3-5 servings/week | ≥6 servings/week |
| Mushroom or algae | <1 servings/week | 1-3 servings/week | ≥4 servings/week |
| Fresh fruit | ≤2 servings/week | 3-5 servings/week | ≥6 servings/week |
| Cooking oil | Animal oil |  | Vegetable oil |
| Fish | <1 meal/month | 1-3 meal/month | ≥1 meal/week |
| Soybeans | <1 meal/month | 1-3 meal/month | ≥1 meal/week |
| Nuts | <1 servings/month | 1-4 servings/month | ≥5 servings/week |
| Garlic | <1 meal/week | 1-3 meal/week | ≥4 meals/week |
| Tea | Not almost every day | Other types of tea (almost every day) | Green tea (almost every day) |
| Sugar or sweets | ≥2 servings/week | 1 serving/month-1 serving/week | <1 serving/month |

## Table S2. The components and scores of the anti-inflammatory dietary diversity

| **Components** | **Score 0** | **Score 1** |
| --- | --- | --- |
| Fresh fruit | "rarely or never" or "occasionally" | "almost every day" or "frequently" |
| Fresh vegetables | "rarely or never" or "occasionally" | "almost every day" or "frequently" |
| Legumes and their products | "rarely or never" or "not every month, but occasionally" or "not every week, but at least once per month" | "almost every day" or "not every day, but at least once per week" |
| Nuts | "rarely or never" or "not every month, but occasionally" or "not every week, but at least once per month" | "almost every day" or "not every day, but at least once per week" |
| Tea | "rarely or never" or "not every month, but occasionally" or "not every week, but at least once per month" | "almost every day" or "not every day, but at least once per week" |

## Table S3. The Generalized Anxiety Disorder (GAD-7) scale and scores

| **No.** | **Items** | **Score 0** | **Score 1** | **Score 2** | **Score 3** |
| --- | --- | --- | --- | --- | --- |
| GAD 1 | Feeling uneasy, worried and annoyed (nervousness or anxiety) | never | for several days | for more than half of days | for almost every day |
| GAD 2 | Can’t stop or can’t control worry (uncontrollable worry) | never | for several days | for more than half of days | for almost every day |
| GAD 3 | Can’t stop or can’t control worry (generalized worry) | never | for several days | for more than half of days | for almost every day |
| GAD 4 | Is very nervous and it is difficult to relax (trouble relaxing) | never | for several days | for more than half of days | for almost every day |
| GAD 5 | Is very anxious, so you can’t sit still (restlessness) | never | for several days | for more than half of days | for almost every day |
| GAD 6 | Becomes easy to get annoyed or easily irritated (irritability) | never | for several days | for more than half of days | for almost every day |
| GAD 7 | Feels like something terrible happens (fear of awful events) | never | for several days | for more than half of days | for almost every day |

## Table S4. Comparison of characteristics in analysis dataset and excluded data

| **Baseline characteristics** | **Analysis dataset**  **（n=13,815)** | **Excluded data**  **(n=1964)** | **SMD^*^** |
| --- | --- | --- | --- |
| Regions |  |  |  |
| North | 822 (6.0) | 112 (5.7) | 0.073 |
| Northeast | 612 (4.4) | 81 (4.1) |  |
| East | 5595 (40.5) | 784 (40.1) |  |
| Central South | 5027 (36.4) | 682 (34.9) |  |
| West | 1759 (12.7) | 297 (15.2) |  |
| Age (years) |  |  |  |
| 65~79 | 5034 (36.4) | 318 (16.3) | **0.673** |
| 80~89 | 3661 (26.5) | 343 (17.5) |  |
| 90~99 | 3000 (21.7) | 538 (27.5) |  |
| ≥100 | 2120 (15.3) | 757 (38.7) |  |
| Sex |  |  |  |
| Male | 6230 (45.1) | 639 (32.7) | **0.257** |
| Female | 7585 (54.9) | 1317 (67.3) |  |
| Ethnicity: Han | 11264 (81.5) | 1535 (78.5) | 0.077 |
| Educated time (year) |  |  |  |
| 0 (Illiteracy) | 5696 (48.1) | 1094 (68.2) | **0.418** |
| 1~6 | 3855 (32.6) | 340 (21.2) |  |
| 7~9 | 1159 (9.8) | 80 (5.0) |  |
| 10~12 | 689 (5.8) | 55 (3.4) |  |
| ≥13 | 433 (3.7) | 36 (2.2) |  |
| Agriculture-based occupation | 7236 (52.4) | 1022 (52.2) | 0.003 |
| Marital status |  |  |  |
| Married, living with spouse | 5695 (41.7) | 370 (20.1) | **0.488** |
| Widow | 7573 (55.4) | 1420 (77.0) |  |
| Divorce or separation | 292 (2.1) | 31 (1.7) |  |
| Never married | 113 (0.8) | 23 (1.2) |  |
| Household registration |  |  |  |
| Urban | 3859 (28.0) | 445 (23.8) | 0.096 |
| Rural | 9902 (72.0) | 1421 (76.2) |  |
| Living arrangements |  |  |  |
| Living with family | 10875 (79.9) | 1540 (83.4) | 0.164 |
| Living alone | 2259 (16.6) | 211 (11.4) |  |
| Collective institutions | 477 (3.5) | 95 (5.1) |  |
| Indoor air pollution ^a^ |  |  |  |
| None | 8205 (59.4) | 1067 (54.6) | 0.101 |
| Level 1 | 4824 (34.9) | 775 (39.6) |  |
| Level 2 | 786 (5.7) | 114 (5.8) |  |
| Economic status ^b^ |  |  |  |
| Rich | 2709 (19.8) | 271 (14.9) | 0.194 |
| Medium | 9557 (69.9) | 1266 (69.4) |  |
| Poor | 1403 (10.3) | 287 (15.7) |  |
| Pension insurance participation | 4999 (36.2) | 698 (35.7) | 0.010 |
| Smoking |  |  |  |
| Never | 8588 (67.0) | 1323 (77.5) | **0.263** |
| Previous | 2006 (15.7) | 226 (13.2) |  |
| Current | 2216 (17.3) | 158 (9.3) |  |
| Drinking |  |  |  |
| Never | 9948 (73.5) | 1464 (81.2) | **0.202** |
| Previous | 1588 (11.7) | 179 (9.9) |  |
| Current | 2007 (14.8) | 160 (8.9) |  |
| Regular physical exercise | 3380 (24.5) | 233 (11.9) | **0.330** |
| BMI (kg/m^2^) |  |  |  |
| <18.5 (Underweight) | 2077 (16.0) | 388 (27.0) | **0.298** |
| 18.5~24.9 (Normal) | 7819 (60.1) | 804 (56.0) |  |
| 25.0~29.9 (Overweight) | 2616 (20.1) | 193 (13.4) |  |
| ≥30.0 (Obese) | 504 (3.9) | 50 (3.5) |  |
| WC>85cm (male); >80cm (female) | 7400 (53.6) | 734 (37.5) | **0.326** |
| Comorbidities |  |  |  |
| Visual impairment | 2135 (15.5) | 1149 (58.7) | **1.002** |
| Hearing impairment | 5219 (37.8) | 1360 (69.5) | **0.672** |
| Toothache or cheek pain | 2426 (17.6) | 201 (10.3) | **0.212** |
| Cardiometabolic diseases ^c^ | 7204 (52.1) | 845 (43.2) | 0.180 |
| Respiratory system diseases ^d^ | 1428 (10.3) | 207 (10.6) | 0.008 |
| Digestive system diseases ^e^ | 1066 (7.7) | 142 (7.3) | 0.017 |
| Immune system diseases ^f^ | 1819 (13.2) | 215 (11.0) | 0.067 |
| Cancer | 179 (1.3) | 25 (1.3) | 0.002 |
| Frailty index >0.25 | 2043 (14.9) | 1178 (67.1) | **1.251** |
| Cognitive impairment ^g^ | 2394 (20.2) | 1055 (65.7) | **1.035** |
| Fall last year | 3070 (22.2) | 503 (25.7) | 0.082 |
| Health changes last year ^h^ |  |  |  |
| Stable | 6696 (50.9) | 495 (44.0) | 0.150 |
| Better | 1740 (13.2) | 149 (13.2) |  |
| Worse | 4714 (35.8) | 482 (42.8) |  |
| Sleep quality ^i^ |  |  |  |
| Good | 6918 (52.4) | 614 (51.2) | 0.081 |
| Neutral | 4291 (32.5) | 369 (30.8) |  |
| Poor | 1984 (15.0) | 216 (18.0) |  |
| Quality of life ^j^ |  |  |  |
| Good | 9262 (70.4) | 757 (66.9) | 0.136 |
| Neutral | 3538 (26.9) | 315 (27.9) |  |
| Poor | 353 (2.7) | 59 (5.2) |  |
| Depression |  |  |  |
| CESD-10 score <12 | 11754 (85.1) | 1748 (89.4) | 0.129 |
| CESD-10 score ≥12 | 2061 (14.9) | 208 (10.6) |  |
| BADL score ^k^, mean (SD) | 6.91 (2.24) | 10.14 (4.36) | **0.932** |
| IADL score ^l^, mean (SD) | 13.32 (5.95) | 19.17 (6.23) | **0.961** |
| Participating solitary activity ^m^ | 11566 (83.7) | 985 (50.4) | **0.759** |
| Participating social activity ^n^ | 13459 (97.4) | 1837 (93.9) | 0.173 |

Abbreviation: BADL: basic activities of daily living; BMI: body mass index; CESD: center for epidemiologic studies depression scale; DD: dietary diversity; GAD: the generalized anxiety disorder scale; IADL: instrumental activity of daily living; SD: standard deviation; SMD: standard mean difference; WC: waist circumference.

Note: Data were shown as absolute frequency and percentage, unless otherwise specified.

^*^ SMD>0.2 indicates the difference between groups is significant.

^a^ Indoor air pollution was defined 0~2 levels by indoor moldy taste and cooking with polluting fuels (e.g., coal, wood, or kerosene).

^b^ Economic status was categorized as "rich", "medium", or "poor" based on the interviewee’s self-assessment relative to others, with "rich" for "very rich" or "rich", "medium" for "so-so", and "poor" for "poor" or "very poor".

^c^ Cardiometabolic diseases included hypertension, diabetes, heart diseases, cerebrovascular diseases or stroke, and dyslipidemia.

^d^ Respiratory system diseases included bronchopneumonia, emphysema, asthma, and tuberculosis.

^e^ Digestive system diseases included gastrointestinal ulcer, cholecystitis, cholelithiasis, and hepatitis.

^f^ Immune system diseases included arthritis and rheumatism.

^g^ Cognitive impairment was identified by the Chinese version of 30-point mini-mental state examination (MMSE) about orientation, memory, attention, calculation, language, and written and visual construction, which defined those scored less than or equal to 17 as cognitive impairment for those without formal education, less than or equal to 20 for those with 1-6 years of education, and less than or equal to 24 for those with more than 6 years of education.

^h^ Health change was categorized as "stable" if the interviewee rated their health as "almost the same" compared to one year ago, "better" if rated as "much better" or "slightly better", and "worse" if rated as "much worse" or "slightly worse".

^i^ Sleep quality was categorized as "good" if the interviewee rated their sleep quality as "good" or "very good", "neutral" if rated as "so-so", and "poor" if rated as "poor" or "very poor".

^j^ Quality of life was categorized as "good" if the interviewee rated their quality of life as "good" or "very good", "neutral" if rated as "so-so", and "poor" if rated as "poor" or "very poor".

^k^ BADL included bathing, dressing, toileting, indoor moving, continence of defecation, and eating, and total scored 6-18 with larger value indicating poorer activities.

^l^ IADL included visiting neighbors, shopping, cooking, washing, continuously walking two kilometers, lifting 5 kilograms objects, squat and stand up three times, or taking public transportation by themselves, and total scored 8-24 with larger value indicating poorer activities.

^m^ Solitary activity included reading newspapers or books, watching TV or listening to radio, doing housework, gardening, and keeping pets or domestic animals.

^n^ Social activity included playing cards or mahjong, attending social activities, participating in outdoor activities, regular exercise, or travelling at least once in the past 2 years.

## Table S5. Univariable analysis of association between the cMIND diet, anti-inflammatory dietary diversity, baseline characteristics and risk of anxiety

| **Diet and baseline characteristics** | **Anxiety** | **Control** | **Crude OR^*^ (95%CI)** | ***p* value^**^** |
| --- | --- | --- | --- | --- |
|  | **(n=1550)** | **(n=12,265)** |  |  |
| Adherence to cMIND ^†^ |  |  |  |  |
| High | 1267 (81.7) | 8824 (71.9) | 1.00 | <0.001 |
| Low | 283 (18.3) | 3441 (28.1) | 0.57 (0.50-0.65) |  |
| Anti-inflammatory DD ^†^ |  |  |  |  |
| High | 1139 (73.5) | 7507 (61.2) | 1.00 | <0.001 |
| Low | 411 (26.5) | 4758 (38.8) | 0.57 (0.51-0.64) |  |
| Regions |  |  |  |  |
| North | 63 (4.1) | 759 (6.2) | 1.00 | <0.001 |
| Northeast | 45 (2.9) | 567 (4.6) | 0.96 (0.64-1.42） |  |
| East | 626 (40.4) | 4969 (40.5) | 1.52 (1.17-2.01） |  |
| Central South | 581 (37.5) | 4446 (36.2) | 1.57 (1.21-2.08） |  |
| West | 235 (15.2) | 1524 (12.4) | 1.86 (1.40-2.50） |  |
| Age (years) |  |  |  |  |
| 65~80 | 626 (40.4) | 4408 (35.9) | 1.00 | <0.001 |
| 80~89 | 430 (27.7) | 3231 (26.3) | 0.94 (0.82-1.07） |  |
| 90~99 | 295 (19.0) | 2705 (22.1) | 0.77 (0.66-0.89） |  |
| ≥100 | 199 (12.8) | 1921 (15.7) | 0.73 (0.62-0.86） |  |
| Sex |  |  |  |  |
| Male | 547 (35.3) | 5683 (46.3) | 1.00 | <0.001 |
| Female | 1003 (64.7) | 6582 (53.7) | 1.58 (1.42-1.77） |  |
| Ethnicity |  |  |  |  |
| Other | 231 (14.9) | 2320 (18.9) | 1.00 | <0.001 |
| Han | 1319 (85.1) | 9945 (81.1) | 1.33 (1.15-1.55） |  |
| Educated time (year) |  |  |  |  |
| 0 (illiteracy) | 878 (56.6) | 5777 (47.1) | 1.00 | <0.001 |
| 1~6 | 452 (29.2) | 4060 (33.1) | 0.73 (0.65-0.83） |  |
| 7~9 | 109 (7.0) | 1265 (10.3) | 0.57 (0.46-0.70） |  |
| 10~12 | 67 (4.3) | 733 (6.0) | 0.60 (0.46-0.77） |  |
| ≥13 | 44 (2.8) | 430 (3.5) | 0.67 (0.48-0.91） |  |
| Occupation |  |  |  |  |
| Other | 636 (41.0) | 5943 (48.5) | 1.00 | <0.001 |
| Agriculture-based | 914 (59.0) | 6322 (51.5) | 1.35 (1.21-1.50） |  |
| Marital status |  |  |  |  |
| Married, living with spouse | 625 (40.3) | 5119 (41.7) | 1.00 | 0.12 |
| Widow | 869 (56.1) | 6795 (55.4) | 1.05 (0.94-1.17） |  |
| Divorce or separation | 36 (2.3) | 258 (2.1) | 1.14 (0.79-1.61） |  |
| Never married | 20 (1.3) | 93 (0.8) | 1.76 (1.05-2.81） |  |
| Household registration |  |  |  |  |
| Urban | 359 (23.2) | 3511 (28.6) | 1.00 | <0.001 |
| Rural | 1191 (76.8) | 8754 (71.4) | 1.33 (1.18-1.51） |  |
| Living arrangements |  |  |  |  |
| Living with family | 1203 (77.6) | 9849 (80.3) | 1.00 | 0.018 |
| Living alone | 295 (19.0) | 1986 (16.2) | 1.22 (1.06-1.39） |  |
| Collective institutions | 52 (3.4) | 430 (3.5) | 0.99 (0.73-1.32） |  |
| Indoor air pollution ^a^ |  |  |  |  |
| None | 745 (48.1) | 7460 (60.8) | 1.00 | <0.001 |
| Level 1 | 640 (41.3) | 4184 (34.1) | 1.53 (1.37-1.71） |  |
| Level 2 | 165 (10.6) | 621 (5.1) | 2.66 (2.20-3.20） |  |
| Economic status ^b^ |  |  |  |  |
| Rich | 169 (10.9) | 2556 (20.8) | 1.00 | <0.001 |
| Medium | 1048 (67.6) | 8626 (70.3) | 1.84 (1.56-2.18） |  |
| Poor | 333 (21.5) | 1083 (8.8) | 4.65 (3.82-5.68） |  |
| Pension insurance participation (ref. none) | 625 (40.3) | 4374 (35.7) | 1.22 (1.09-1.36） | <0.001 |
| Smoking |  |  |  |  |
| Never | 1154 (74.5) | 8280 (67.5) | 1.00 | <0.001 |
| Previous | 188 (12.1) | 1888 (15.4) | 0.71 (0.61-0.84） |  |
| Current | 208 (13.4) | 2097 (17.1) | 0.71 (0.61-0.83） |  |
| Drinking |  |  |  |  |
| Never | 1195 (77.1) | 8993 (73.3) | 1.00 | <0.001 |
| Previous | 184 (11.9) | 1414 (11.5) | 0.98 (0.83-1.15） |  |
| Current | 171 (11.0) | 1858 (15.1) | 0.69 (0.58-0.82） |  |
| Regular physical activity (ref. none) | 329 (21.2) | 3051 (24.9) | 0.81 (0.71-0.92） | 0.002 |
| Body mass index (kg/m^2^) |  |  |  |  |
| 18.5~24.9 (normal) | 926 (59.7) | 7389 (60.2) | 1.00 | <0.001 |
| <18.5 (underweight) | 304 (19.6) | 1979 (16.1) | 1.23 (1.07-1.41） |  |
| 25.0~29.9 (overweight) | 255 (16.5) | 2435 (19.9) | 0.84 (0.72-0.97） |  |
| ≥30.0 (obese) | 65 (4.2) | 462 (3.8) | 1.12 (0.85-1.46） |  |
| Waist circumference |  |  |  |  |
| ≤85cm (male); ≤80cm (female) | 783 (50.5) | 5632 (45.9) | 1.00 | 0.001 |
| >85cm (male); >80cm (female) | 767 (49.5) | 6633 (54.1) | 0.83 (0.75-0.92） |  |
| Comorbidities |  |  |  |  |
| Visual impairment (ref. none) | 320 (20.6) | 1815 (14.8) | 1.50 (1.31-1.71） | <0.001 |
| Hearing impairment (ref. none) | 643 (41.5) | 4576 (37.3) | 1.19 (1.07-1.33） | 0.002 |
| Toothache or cheek pain (ref. none) | 456 (29.4) | 1970 (16.1) | 2.18 (1.93-2.45） | <0.001 |
| Cardiometabolic diseases ^c^ (ref. none) | 882 (56.9) | 6322 (51.5) | 1.24 (1.12-1.38） | <0.001 |
| Respiratory system diseases ^d^ (ref. none) | 201 (13.0) | 1227 (10.0) | 1.34 (1.14-1.57） | <0.001 |
| Digestive system diseases ^e^ (ref. none) | 169 (10.9) | 897 (7.3) | 1.55 (1.30-1.84） | <0.001 |
| Immune system diseases ^f^ (ref. none) | 280 (18.1) | 1539 (12.5) | 1.54 (1.33-1.76） | <0.001 |
| Cancer (ref. none) | 28 (1.8) | 151 (1.2) | 1.48 (0.96-2.18） | 0.077 |
| Frailty index |  |  |  |  |
| ≤0.25 | 1152 (74.3) | 10556 (86.1) | 1.00 | <0.001 |
| >0.25 | 398 (25.7) | 1709 (13.9) | 2.13 (1.88-2.42） |  |
| Cognitive impairment ^g^ (ref. none) | 322 (20.8) | 2319 (18.9) | 1.12 (0.99-1.28） | 0.08 |
| Fall last year (ref. none) | 533 (34.4) | 2537 (20.7) | 2.01 (1.79-2.25） | <0.001 |
| Health changes last year ^h^ |  |  |  |  |
| Stable | 522 (33.7) | 6538 (53.3) | 1.00 | <0.001 |
| Better | 168 (10.8) | 1640 (13.4) | 1.28 (1.07-1.54） |  |
| Worse | 860 (55.5) | 4087 (33.3) | 2.64 (2.35-2.96） |  |
| Sleep quality ^i^ |  |  |  |  |
| Good | 407 (26.3) | 6902 (56.3) | 1.00 | <0.001 |
| Neutral | 579 (37.4) | 3891 (31.7) | 2.52 (2.21-2.88） |  |
| Poor | 564 (36.4) | 1472 (12.0) | 6.50 (5.65-7.47） |  |
| Quality of life ^j^ |  |  |  |  |
| Good | 804 (51.9) | 9003 (73.4) | 1.00 | <0.001 |
| Neutral | 600 (38.7) | 3037 (24.8) | 2.21 (1.97-2.48） |  |
| Poor | 146 (9.4) | 225 (1.8) | 7.27 (5.82-9.05） |  |
| CESD-10 score |  |  |  |  |
| <12 | 716 (46.2) | 11038 (90.0) | 1.00 | <0.001 |
| ≥12 (depression) | 834 (53.8) | 1227 (10.0) | 10.48 (9.33-11.77） |  |
| BADL score ^k^, mean (SD) | 7.07 (2.43) | 6.89 (2.21) | 1.03 (1.01-1.06） | 0.003 |
| IADL score ^l^, mean (SD) | 14.00 (5.91) | 13.23 (5.94) | 1.02 (1.01-1.03） | <0.001 |
| Participating solitary activity ^m^ (ref. none) | 1262 (81.4) | 10304 (84.0) | 0.83 (0.73-0.96） | 0.01 |
| Participating social activity ^n^ (ref. none) | 1515 (97.7) | 11944 (97.4) | 1.16 (0.83-1.68） | 0.45 |

Abbreviation: BADL: basic activities of daily living; CESD: center for epidemiologic studies depression scale; DD: dietary diversity; IADL: instrumental activity of daily living; SD: standard deviation.

Note: Data were shown as absolute frequency and percentage, unless otherwise specified.

^*^ Univariable logistic regression. ^**^Chi-square test or *t* test. ^†^The criteria for high adherence to the cMIND and AIDD diets were defined as scores exceeding the upper quartile cut-off, while scores at or below this threshold were classified as low adherence.

^a^ Indoor air pollution was defined 0~2 levels by indoor moldy taste and cooking with polluting fuels (e.g., coal, wood, or kerosene).

^b^ Economic status was categorized as "rich", "medium", or "poor" based on the interviewee’s self-assessment relative to others, with "rich" for "very rich" or "rich", "medium" for "so-so", and "poor" for "poor" or "very poor".

^c^ Cardiometabolic diseases included hypertension, diabetes, heart diseases, cerebrovascular diseases or stroke, and dyslipidemia.

^d^ Respiratory system diseases included bronchopneumonia, emphysema, asthma, and tuberculosis.

^e^ Digestive system diseases included gastrointestinal ulcer, cholecystitis, cholelithiasis, and hepatitis.

^f^ Immune system diseases included arthritis and rheumatism.

^g^ Cognitive impairment was identified by the Chinese version of 30-point mini-mental state examination (MMSE) about orientation, memory, attention, calculation, language, and written and visual construction, which defined those scored less than or equal to 17 as cognitive impairment for those without formal education, less than or equal to 20 for those with 1-6 years of education, and less than or equal to 24 for those with more than 6 years of education.

^h^ Health change was categorized as "stable" if the interviewee rated their health as "almost the same" compared to one year ago, "better" if rated as "much better" or "slightly better", and "worse" if rated as "much worse" or "slightly worse".

^i^ Sleep quality was categorized as "good" if the interviewee rated their sleep quality as "good" or "very good", "neutral" if rated as "so-so", and "poor" if rated as "poor" or "very poor".

^j^ Quality of life was categorized as "good" if the interviewee rated their quality of life as "good" or "very good", "neutral" if rated as "so-so", and "poor" if rated as "poor" or "very poor".

^k^ BADL included bathing, dressing, toileting, indoor moving, continence of defecation, and eating, and total scored 6-18 with larger value indicating poorer activities.

^l^ IADL included visiting neighbors, shopping, cooking, washing, continuously walking two kilometers, lifting 5 kilograms objects, squat and stand up three times, or taking public transportation by themselves, and total scored 8-24 with larger value indicating poorer activities.

^m^ Solitary activity included reading newspapers or books, watching TV or listening to radio, doing housework, gardening, and keeping pets or domestic animals.

^n^ Social activity included playing cards or mahjong, attending social activities, participating in outdoor activities, regular exercise, or travelling at least once in the past 2 years.

| 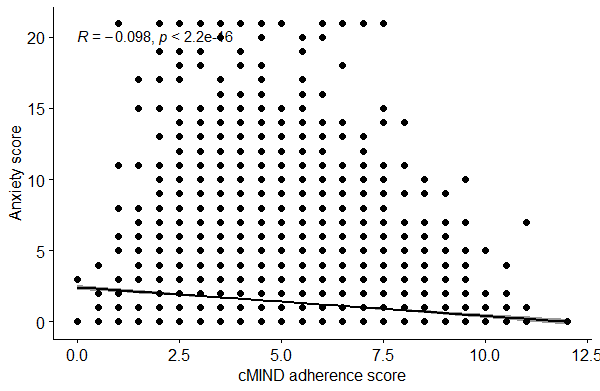Figure S1. The spearman correlation analysis between cMIND adherence score and anxiety score |
| --- |
| 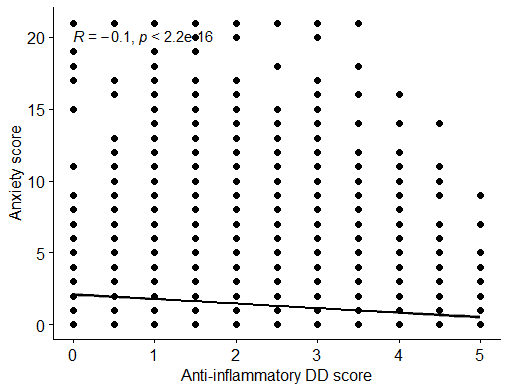Figure S2. The spearman correlation analysis between ant-inflammatory dietary diversity (DD) score and anxiety score |

## Table S6. Multivariable logistic regression of anxiety and all variables in models (n=13,815)

| **Variables in model** | **Model 1** | **Model 2** | **Model 3** | **Model 4** | **Model 1** | **Model 2** | **Model 3** | **Model 4** |
| --- | --- | --- | --- | --- | --- | --- | --- | --- |
|  | **aOR (95%CI）** | **aOR (95%CI）** | **aOR (95%CI）** | **aOR (95%CI）** | **aOR (95%CI）** | **aOR (95%CI）** | **aOR (95%CI）** | **aOR (95%CI）** |
| Adherence to cMIND: high vs. low (ref.) | **0.75 (0.64-0.87)** | **0.82 (0.70-0.96)** | **0.84 (0.71-0.99)** | **0.75 (0.61-0.91)** | —— | —— | —— | —— |
| Anti-inflammatory DD: high vs. low (ref.) | —— | —— | —— | —— | **0.75 (0.66-0.86)** | **0.82 (0.72-0.94)** | **0.84 (0.73-0.96)** | **0.74 (0.62-0.88)** |
| Regions (ref. north) |  |  |  |  |  |  |  |  |
| Northeast | 0.76 (0.50-1.16) | 0.94 (0.61-1.43) | 0.91 (0.58-1.41) | 0.92 (0.59-1.43) | 0.75 (0.49-1.13) | 0.93 (0.60-1.41) | 0.90 (0.58-1.39) | 0.91 (0.58-1.41) |
| East | 1.15 (0.87-1.55) | 1.17 (0.88-1.59) | 1.01 (0.75-1.38) | 1.00 (0.74-1.37) | 1.13 (0.85-1.53) | 1.15 (0.86-1.56) | 1.00 (0.74-1.37) | 0.99 (0.73-1.36) |
| Central South | 1.15 (0.86-1.56) | 1.06 (0.79-1.45) | 0.93 (0.69-1.29) | 0.93 (0.68-1.29) | 1.13 (0.84-1.53) | 1.04 (0.77-1.42) | 0.92 (0.67-1.27) | 0.91 (0.67-1.26) |
| West | 1.21 (0.89-1.67) | 1.17 (0.85-1.63) | 1.19 (0.85-1.67) | 1.18 (0.85-1.66) | 1.18 (0.87-1.63) | 1.15 (0.84-1.60) | 1.17 (0.84-1.64) | 1.16 (0.83-1.63) |
| Age (ref. 65~80 years) |  |  |  |  |  |  |  |  |
| 80~89 years | **0.76 (0.65-0.88)** | **0.75 (0.64-0.87)** | **0.72 (0.61-0.85)** | **0.72 (0.61-0.85)** | **0.76 (0.66-0.89)** | **0.75 (0.64-0.87)** | **0.72 (0.61-0.85)** | **0.72 (0.62-0.85)** |
| 90~99 years | **0.56 (0.46-0.67)** | **0.60 (0.50-0.73)** | **0.60 (0.49-0.73)** | **0.60 (0.50-0.73)** | **0.56 (0.47-0.68)** | **0.61 (0.50-0.73)** | **0.60 (0.50-0.74)** | **0.61 (0.50-0.74)** |
| ≥100 years | **0.43 (0.34-0.54)** | **0.49 (0.39-0.62)** | **0.55 (0.43-0.69)** | **0.55 (0.43-0.70)** | **0.43 (0.35-0.54)** | **0.50 (0.40-0.62)** | **0.55 (0.44-0.70)** | **0.55 (0.44-0.70)** |
| Sex: female vs. male (ref.) | **1.27 (1.09-1.49)** | **1.21 (1.03-1.41)** | **1.18 (1.00-1.39)** | **1.18 (1.00-1.39)** | **1.28 (1.09-1.49)** | **1.21 (1.03-1.41)** | **1.18 (1.00-1.39)** | **1.18 (1.00-1.39)** |
| Ethnicity: Han vs. other (ref.) | **1.32 (1.11-1.56)** | **1.29 (1.09-1.54)** | **1.31 (1.09-1.57)** | **1.31 (1.09-1.57)** | **1.32 (1.11-1.57)** | **1.29 (1.09-1.54)** | **1.31 (1.09-1.57)** | **1.31 (1.09-1.57)** |
| Educated time (ref. 0 year) | | | | | | | | |
| 1~6 years | **0.82 (0.71-0.94)** | **0.83 (0.72-0.96)** | **0.84 (0.72-0.98)** | **0.84 (0.72-0.98)** | **0.81 (0.70-0.93)** | **0.83 (0.72-0.96)** | **0.84 (0.72-0.97)** | **0.83 (0.72-0.97)** |
| 7~9 years | **0.72 (0.56-0.92)** | **0.73 (0.57-0.93)** | **0.75 (0.58-0.96)** | **0.75 (0.58-0.97)** | **0.72 (0.56-0.91)** | **0.72 (0.56-0.92)** | **0.74 (0.57-0.96)** | **0.75 (0.58-0.96)** |
| 10~12 years | 0.83 (0.61-1.11) | 0.82 (0.60-1.11) | 0.79 (0.57-1.09) | 0.79 (0.57-1.09) | 0.82 (0.61-1.10) | 0.81 (0.60-1.10) | 0.79 (0.57-1.08) | 0.79 (0.57-1.09) |
| ≥13 years | 0.90 (0.61-1.28) | 0.87 (0.59-1.26) | 0.82 (0.54-1.20) | 0.82 (0.54-1.20) | 0.90 (0.62-1.29) | 0.88 (0.59-1.27) | 0.82 (0.55-1.21) | 0.82 (0.54-1.20) |
| Occupation: agriculture-based vs. other (ref.) | 1.00 (0.87-1.15) | 1.00 (0.87-1.16) | 1.03 (0.89-1.20) | 1.03 (0.89-1.20) | 1.00 (0.87-1.15) | 1.00 (0.87-1.15) | 1.03 (0.89-1.20) | 1.03 (0.89-1.20) |
| Marital status (ref. married, living with spouse) | | | | | | | | |
| Widow | 1.03 (0.88-1.20) | 1.05 (0.90-1.23) | 0.98 (0.83-1.15) | 0.98 (0.83-1.15) | 1.03 (0.89-1.21) | 1.06 (0.90-1.24) | 0.98 (0.83-1.15) | 0.98 (0.83-1.15) |
| Divorce or separation | 1.12 (0.75-1.63) | 1.16 (0.77-1.71) | 1.09 (0.70-1.64) | 1.08 (0.69-1.62) | 1.11 (0.74-1.61) | 1.16 (0.76-1.70) | 1.08 (0.70-1.63) | 1.08 (0.70-1.63) |
| Never married | 1.37 (0.79-2.30) | 1.56 (0.88-2.65) | 1.24 (0.67-2.20) | 1.23 (0.67-2.18) | 1.37 (0.78-2.30) | 1.56 (0.88-2.66) | 1.24 (0.67-2.20) | 1.24 (0.67-2.20) |
| Household registration: rural vs. urban (ref.) | 1.02 (0.86-1.21) | 1.07 (0.90-1.28) | 1.12 (0.93-1.34) | 1.12 (0.93-1.34) | 1.01 (0.85-1.19) | 1.07 (0.90-1.27) | 1.11 (0.92-1.33) | 1.11 (0.93-1.34) |
| Living arrangements (ref. living with family) | | | | | | | | |
| Living alone | 1.03 (0.87-1.21) | 0.94 (0.80-1.11) | 0.89 (0.74-1.06) | 0.90 (0.75-1.07) | 1.03 (0.87-1.21) | 0.94 (0.80-1.11) | 0.89 (0.74-1.06) | 0.89 (0.75-1.06) |
| Collective institutions | 0.84 (0.60-1.16) | 0.78 (0.55-1.10) | 0.79 (0.55-1.13) | 0.78 (0.54-1.12) | 0.84 (0.60-1.16) | 0.78 (0.55-1.09) | 0.79 (0.55-1.13) | 0.79 (0.54-1.12) |
| Indoor air pollution ^a^ (ref. none) | | | | | | | | |
| Level 1 | **1.28 (1.13-1.45)** | **1.21 (1.07-1.38)** | **1.16 (1.01-1.34)** | **1.16 (1.01-1.33)** | **1.28 (1.13-1.46)** | **1.22 (1.07-1.39)** | **1.17 (1.02-1.34)** | **1.16 (1.01-1.33)** |
| Level 2 | **1.69 (1.37-2.08)** | **1.52 (1.22-1.88)** | **1.37 (1.09-1.72)** | **1.38 (1.10-1.73)** | **1.69 (1.37-2.08)** | **1.52 (1.22-1.88)** | **1.37 (1.09-1.72)** | **1.38 (1.10-1.73)** |
| Economic status ^b^ (ref. rich) | | | | | | | | |
| Medium | **1.46 (1.23-1.74)** | **1.22 (1.02-1.47)** | 1.13 (0.94-1.36) | 1.12 (0.93-1.36) | **1.45 (1.22-1.74)** | **1.22 (1.02-1.47)** | 1.12 (0.94-1.36) | 1.12 (0.93-1.35) |
| Poor | **2.61 (2.10-3.25)** | **1.66 (1.32-2.10)** | **1.37 (1.07-1.75)** | **1.37 (1.08-1.75)** | **2.58 (2.08-3.21)** | **1.64 (1.30-2.08)** | **1.36 (1.06-1.73)** | **1.36 (1.07-1.74)** |
| Pension insurance participation: yes vs. no (ref.) | **1.12 (1.00-1.26)** | **1.16 (1.03-1.30)** | **1.21 (1.07-1.37)** | **1.21 (1.07-1.37)** | **1.13 (1.00-1.27)** | **1.16 (1.03-1.31)** | **1.21 (1.07-1.38)** | **1.22 (1.07-1.38)** |
| Smoking (ref. never) |  |  |  |  |  |  |  |  |
| Previous | **0.81 (0.66-0.99)** | 0.82 (0.67-1.01) | 0.86 (0.70-1.06) | 0.86 (0.69-1.06) | **0.81 (0.66-0.99)** | 0.82 (0.67-1.01) | 0.86 (0.70-1.06) | 0.86 (0.70-1.06) |
| Current | 0.83 (0.69-1.01) | 0.86 (0.71-1.05) | 0.85 (0.69-1.04) | 0.85 (0.69-1.04) | 0.83 (0.69-1.01) | 0.87 (0.71-1.05) | 0.85 (0.70-1.05) | 0.85 (0.69-1.04) |
| Drinking (ref. never) |  |  |  |  |  |  |  |  |
| Previous | 1.09 (0.90-1.31) | 1.10 (0.91-1.34) | 1.05 (0.85-1.29) | 1.05 (0.85-1.29) | 1.08 (0.89-1.31) | 1.10 (0.90-1.34) | 1.04 (0.85-1.28) | 1.05 (0.85-1.29) |
| Current | 0.93 (0.77-1.13) | 0.95 (0.78-1.16) | 0.95 (0.77-1.17) | 0.95 (0.78-1.17) | 0.94 (0.77-1.13) | 0.96 (0.79-1.16) | 0.96 (0.78-1.17) | 0.95 (0.78-1.17) |
| Regular physical activity: yes vs. no (ref.) | 1.08 (0.93-1.24) | 1.12 (0.96-1.29) | **1.21 (1.03-1.41)** | **1.21 (1.04-1.41)** | 1.08 (0.93-1.24) | 1.12 (0.97-1.29) | **1.21 (1.03-1.41)** | **1.21 (1.04-1.41)** |
| BMI (ref. 18.5~24.9 kg/m^2^) | | | | | | | | |
| <18.5 kg/m^2^ | 1.11 (0.95-1.29) | 1.06 (0.90-1.24) | 1.07 (0.90-1.26) | 1.07 (0.91-1.27) | 1.11 (0.95-1.29) | 1.06 (0.91-1.25) | 1.07 (0.91-1.27) | 1.07 (0.91-1.27) |
| 25.0~29.9 kg/m^2^ | 0.84 (0.71-0.98) | 0.87 (0.74-1.02) | 0.87 (0.73-1.04) | 0.87 (0.73-1.04) | **0.84 (0.71-0.98)** | 0.87 (0.74-1.02) | 0.87 (0.73-1.04) | 0.87 (0.73-1.04) |
| ≥30.0 kg/m^2^ | 1.13 (0.84-1.49) | 1.14 (0.85-1.52) | 1.13 (0.83-1.54) | 1.13 (0.82-1.53) | 1.12 (0.83-1.48) | 1.14 (0.84-1.51) | 1.13 (0.82-1.53) | 1.12 (0.82-1.52) |
| WC >85cm (male); >80cm (female): yes vs. no (ref.) | 0.89 (0.79-1.01) | 0.89 (0.78-1.01) | 0.90 (0.79-1.03) | 0.91 (0.79-1.04) | 0.89 (0.78-1.01) | 0.89 (0.78-1.01) | 0.90 (0.79-1.03) | 0.90 (0.79-1.04) |
| Toothache or cheek pain: yes vs. no (ref.) | **1.69 (1.48-1.92)** | **1.54 (1.34-1.76)** | **1.45 (1.26-1.68)** | **1.45 (1.26-1.67)** | **1.67 (1.46-1.90)** | **1.52 (1.33-1.74)** | **1.44 (1.25-1.66)** | **1.45 (1.25-1.67)** |
| Cardiometabolic diseases ^c^: yes vs. no (ref.) | 1.07 (0.95-1.21) | 1.01 (0.90-1.15) | 1.02 (0.90-1.16) | 1.02 (0.90-1.16) | 1.07 (0.95-1.20) | 1.01 (0.89-1.14) | 1.02 (0.89-1.16) | 1.02 (0.89-1.16) |
| Respiratory system diseases ^d^: yes vs. no (ref.) | 1.09 (0.91-1.29) | 1.02 (0.85-1.22) | 0.96 (0.79-1.16) | 0.95 (0.79-1.15) | 1.09 (0.91-1.29) | 1.02 (0.85-1.22) | 0.96 (0.79-1.16) | 0.96 (0.79-1.15) |
| Digestive system diseases ^e^: yes vs. no (ref.) | 1.14 (0.94-1.38) | 1.05 (0.87-1.28) | 1.04 (0.85-1.28) | 1.04 (0.85-1.28) | 1.15 (0.94-1.38) | 1.06 (0.87-1.28) | 1.04 (0.85-1.28) | 1.04 (0.85-1.28) |
| Immune system diseases ^f^: yes vs. no (ref.) | 1.14 (0.98-1.33) | 1.04 (0.89-1.22) | 0.96 (0.81-1.14) | 0.96 (0.81-1.14) | 1.14 (0.98-1.33) | 1.04 (0.89-1.22) | 0.96 (0.82-1.14) | 0.96 (0.82-1.14) |
| Cancer: yes vs. no (ref.) | 1.23 (0.79-1.87) | 1.19 (0.75-1.83) | 1.23 (0.75-1.95) | 1.22 (0.74-1.95) | 1.22 (0.78-1.84) | 1.18 (0.74-1.82) | 1.22 (0.74-1.94) | 1.21 (0.74-1.93) |
| Frailty index: >0.25 vs. ≤0.25 (ref.) | **1.88 (1.61-2.19)** | **1.68 (1.44-1.96)** | **1.36 (1.15-1.60)** | **1.36 (1.15-1.60)** | **1.89 (1.62-2.19)** | **1.68 (1.44-1.97)** | **1.36 (1.15-1.60)** | **1.36 (1.15-1.60)** |
| Fall last year: yes vs. no (ref.) | **1.53 (1.35-1.73)** | **1.48 (1.30-1.68)** | **1.46 (1.28-1.67)** | **1.46 (1.28-1.66)** | **1.53 (1.35-1.73)** | **1.48 (1.30-1.68)** | **1.46 (1.28-1.67)** | **1.46 (1.27-1.66)** |
| Health changes last year ^g^ (ref. stable) | | | | | | | | |
| Better | 1.19 (0.98-1.43) | **1.26 (1.03-1.52)** | 1.21 (0.98-1.47) | 1.21 (0.99-1.47) | 1.19 (0.98-1.43) | **1.25 (1.03-1.52)** | 1.20 (0.98-1.47) | 1.21 (0.99-1.47) |
| Worse | **2.00 (1.77-2.26)** | **1.67 (1.47-1.90)** | **1.36 (1.19-1.56)** | **1.36 (1.19-1.56)** | **1.99 (1.76-2.26)** | **1.67 (1.47-1.90)** | **1.36 (1.19-1.56)** | **1.36 (1.19-1.56)** |
| Sleep quality ^h^ (ref. good) | | | | | | | | |
| Neutral | —— | **1.94 (1.69-2.23)** | **1.46 (1.26-1.69)** | **1.45 (1.25-1.68)** | —— | **1.93 (1.68-2.22)** | **1.45 (1.26-1.69)** | **1.45 (1.25-1.68)** |
| Poor | —— | **3.89 (3.34-4.52)** | **2.44 (2.07-2.87)** | **2.43 (2.07-2.87)** | —— | **3.88 (3.33-4.51)** | **2.44 (2.07-2.87)** | **2.42 (2.06-2.85)** |
| Quality of life ^i^ (ref. good) | | | | | | | | |
| Neutral | —— | **1.42 (1.25-1.62)** | **1.15 (1.00-1.32)** | **1.15 (1.00-1.31)** | —— | **1.42 (1.25-1.62)** | **1.15 (1.00-1.32)** | **1.15 (1.00-1.31)** |
| Poor | —— | **2.77 (2.14-3.57)** | **1.69 (1.28-2.22)** | **1.69 (1.28-2.22)** | —— | **2.77 (2.14-3.57)** | **1.69 (1.28-2.22)** | **1.69 (1.28-2.22)** |
| CESD-10 score ≥12 (depression) vs. <12 (ref.) | —— | —— | **6.15 (5.37-7.04)** | **5.79 (5.01-6.71)** | —— | —— | **6.15 (5.37-7.04)** | **5.62 (4.82-6.55)** |
| Interaction term: diet × depression | —— | —— | —— | **1.39 (1.02-1.91)** | —— | —— | —— | **1.40 (1.07-1.84)** |
| ***AIC of fitted model*** | ***8830.517*** | ***8415.923*** | ***7730.342*** | ***7728.072*** | ***8827.4*** | ***8414.095*** | ***7728.692*** | ***7724.793*** |

Abbreviation: BMI: body mass index; CESD: center for epidemiologic studies depression scale; CI: confidence interval; DD: dietary diversity; OR: adjusted odds ratio; WC: waist circumference.

Note: OR (95% CI) in bold indicates statistical significance.

^a^ Indoor air pollution was defined 0~2 levels by indoor moldy taste and cooking with polluting fuels (e.g., coal, wood, or kerosene).

^b^ Economic status was categorized as "rich", "medium", or "poor" based on the interviewee’s self-assessment relative to others, with "rich" for "very rich" or "rich", "medium" for "so-so", and "poor" for "poor" or "very poor".

^c^ Cardiometabolic diseases included hypertension, diabetes, heart diseases, cerebrovascular diseases or stroke, and dyslipidemia.

^d^ Respiratory system diseases included bronchopneumonia, emphysema, asthma, and tuberculosis.

^e^ Digestive system diseases included gastrointestinal ulcer, cholecystitis, cholelithiasis, and hepatitis.

^f^ Immune system diseases included arthritis and rheumatism.

^g^ Health change was categorized as "stable" if the interviewee rated their health as "almost the same" compared to one year ago, "better" if rated as "much better" or "slightly better", and "worse" if rated as "much worse" or "slightly worse".

^h^ Sleep quality was categorized as "good" if the interviewee rated their sleep quality as "good" or "very good", "neutral" if rated as "so-so", and "poor" if rated as "poor" or "very poor".

^i^ Quality of life was categorized as "good" if the interviewee rated their quality of life as "good" or "very good", "neutral" if rated as "so-so", and "poor" if rated as "poor" or "very poor".

Model 1 adjusted for regions, age, sex, ethnic group, educated time, occupation, marital status, household registration, living pattern, indoor air pollution, economic status, pension insurance, smoking, drinking, regular physical exercise, BMI, waist circumference, toothache or cheek pain, cardiometabolic diseases, respiratory system diseases, digestive system diseases, immune system diseases, cancer, frailty, fall last year, and health change last year.

Model 2 adjusted for the same confounders as model 1, added sleep quality and quality of life.

Model 3 adjusted for the same confounders as model 2, added depression.

Model 4 adjusted for the same confounders as model 3, added the interaction term between diet and depression.
